# Undergraduate Nursing Students’ Willingness of Providing Care for Older Adults With Dementia as Their Future Work

**DOI:** 10.1093/geroni/igab046.256

**Published:** 2021-12-17

**Authors:** Wenlin Liu, Jing Wang

**Affiliations:** Fudan University, shanghai, Shanghai, China (People's Republic)

## Abstract

This study examines how undergraduate nursing students’ knowledge of dementia care are associated with their willingness of providing care for older adults with dementia across care settings as nurses in urban China, controlling for factors such as their socio-demographic characteristics, willingness of being a nurse, and years of studying nursing. We surveyed 320 undergraduate students from Shanghai, China and found that students with a better knowledge of dementia care, a longer year of nursing study, have no experience of being cared for by grandparents during childhood, and being the only child at home tended to be less willing to provide care for older adults with dementia in their future work. In order to prepare high-quality future dementia care workforce, nursing educators not only need to disseminate knowledge of dementia care, they should also tailor teaching to students’ characteristics and motivate students to take the leadership in dementia care across settings.

